# Exposure to Sublethal Ammonia Concentrations Alters the Duration and Intensity of Agonistic Interactions in the Crayfish, *Orconectes rusticus*

**DOI:** 10.1007/s00128-017-2190-7

**Published:** 2017-11-16

**Authors:** David D. Edwards, Katie L. Klotz, Paul A. Moore

**Affiliations:** Laboratory for Sensory Ecology, Department of Biological Sciences, Bowling Green State University, Bowling Green, OH 43403 USA

**Keywords:** Chemosensory, Crayfish, Ammonia, Agonistic interaction

## Abstract

Crayfish extract information from chemical stimuli during social interactions. Commercial fertilizers increase background ammonia concentrations which may interfere with chemical communication. Background pollution can disrupt perception of chemical stimuli in three ways: masking, sensory impairment, physiological impairment or in combination. We investigated whether exposure to ammonia alters agonistic behavior. Crayfish pairs exposed to 0.9 mg/L ammonia fought for a longer duration, while crayfish exposed to 9.0 mg/L ammonia fought for a shorter duration. Altering activity patterns of crayfish may alter crayfish populations leading to a nonproportional impact because of their importance to the structure and function of aquatic ecosystems.

Due to the limited nature of light (visual stimuli) in aquatic habitats, ecological behaviors such as foraging (Weissburg and Zimmer-Faust 1994), locating mates or conspecifics (Hazlett 1985; Endler 1987; Aquiloni et al. 2012), and avoiding predation (Gelowitz et al. 1993) are highly mediated through chemical stimuli (Moore and Crimaldi 2004; Wolf et al. 2009). Chemical stimuli contain information evoking behavioral responses (Jurcak and Moore 2014) which have evolved in habitats free of interference with chemoreception (Endler 2000). However, background environmental pollution can interfere with perception of ecological information in three ways. One way, known as masking, occurs when background chemicals alter the signal to noise ratio of transmitted information changing the ability of the receiver organism to extract relevant information (Dusenbury 1992). Second, chemicals can physiologically impair chemosensory systems by temporarily reducing sensory receptor functions through agonistic / antagonistic binding of chemical molecules to neuroreceptor sites (Sutterlin 1974). Third, background pollution can alter physiological processes in the organism hindering the internal processing of information. A large source of background pollution is through anthropogenic nonpoint source pollution runoff into streams and rivers.

Commercial fertilizers such as anhydrous ammonia and urea are commonly lost during runoff events as they enter nearby streams and other aquatic systems (Eddy 2005). Crustaceans are physiologically impacted by ammonia exposure, including toxicity and death (Arthur et al. 1987; Young-Lai et al. 1991; Romano and Zeng 2010). Ammonia uptake also alters ion regulation in crayfish (Harris et al. 2001). However, crayfish extract important socio-ecological information from chemical stimuli in urine of conspecifics (Zulandt-Schneider et al. 2001; Breithaupt and Eger 2002; Bergman and Moore 2005a, b).

Crayfish urine is important during agonistic behavior while fighting over available resources (Wofford et al. 2015). Dominant crayfish are known to urinate more than subordinate opponents during fights (Bergman et al. 2005). When *Orconectes rusticus* is exposed to urine stimuli from a dominant conspecific prior to fighting, the crayfish will reduce aggression during the fight. When *O. rusticus* is exposed to urine stimuli from a subordinate conspecific prior to fighting, the crayfish will increase aggression during the fight (Bergman and Moore 2005b). Urine release by *O. rusticus* during agonistic battles correlates with decreased time and intensity of subsequent fights with the same conspecific (Zulandt-Schneider et al. 2001). Thus, urine appears to play a role in the assessment strategy used by individuals when deciding to prolong an agonistic interaction (Wofford et al. 2015). The decision to prolong a fight can hinder subsequent behaviors (i.e. —mating, foraging) because of energy expenditure, damage to appendages, or other costs outweighing the acquisition of a resource. Therefore, perception of information from conspecifics is important and could be altered in the presence of background ammonia. The natural habitat of *O. rusticus* for this study in the Portage River, Ohio, USA, fluctuates in ammonia levels from 1.9 × 10 ^−5^ M NH_3_ to 5.3 × 10 ^−5^ M NH_3_ (0.323 mg/L NH_3_ to 0.901 mg/L NH_3_) following peak fertilizing times (Ohio Environmental Protection Agency, OEPA 2010). We hypothesized an elevated ammonia exposure prior to, and during agonistic fights would result in escalated aggression and reduced fight duration when compared to low or no ammonia exposure.

## Materials and Methods

Sixty male, form I *O. rusticus* were collected from the Portage River, Bowling Green, Ohio, USA. Crayfish were grouped into small (carapace: 2.4 ± 0.1 cm, chelae: 1.9 ± 0.1 cm, weight: 5.7 ± 0.4 g; mean ± SEM) and large (carapace: 3.3 ± 0.1 cm, chelae: 3.0 ± 0.1 cm, weight: 13.4 ± 0.8 g; mean ± SEM) sizes. Animals were placed in isolated containers in a flow—through holding tank with a constant temperature (23.1°C) one week before the experiment to remove individual social information and hierarchy status from their natural habitat. Animals were retained in a light: dark cycle (12:12 h) and fed one rabbit pellet three times a week. Crayfish were paired by size - matching with one crayfish at least 30% larger than the opponent to predict a dominance hierarchy (Pavey and Fielder 1996). To identify individuals of the fighting pair, crayfish were uniquely identified (ID) by marking on the carapace with White—Out^®^.

Thirty fighting pairs were equally distributed (N = 10) amongst a control (dechlorinated tap water), ‘low’ (0.9 mg/L NH_3_), and ‘high’ (9.0 mg/L NH_3_) ammonia treatments. The low concentration of 0.9 mg/L NH_3_ was determined by ammonia runoff as reported for the Portage River (Ohio Environmental Protection Agency 2010). The high concentration of 9.0 mg/L NH_3_ was chosen to test for an increased magnitude effect of ammonia on behavior. Both concentrations are considered well below the reported 96-h LC_50_ concentration (300–1000 mg/L NH_3_) for freshwater *Orconectes* crayfish (Arthur et al. 1987). A stock solution of 90 mg/L NH_3_ was prepared using dechlorinated tap water (pH 7.8, dissolved oxygen = 8.32 mg/L, temperature = 23.1°C, hardness = 250 mg/L) and liquid anhydrous ammonia (NH_3_, Sigma-Aldrich). Tanks were aerated to maintain a consistent dissolved oxygen (8.32 mg/L) level and maintained consistent room temperature (23.1°C) throughout the experiment. Ammonia concentrations were verified using a Bausch and Lomb^®^ Spectrophotometer (Spectronic 20) and Hach (Loveland, Colorado, USA) TNTplus^®^, kit TNT831, method 10205. This method is an approved U.S. Environmental Protection Agency (USEPA 350.1 method) equivalent test. Test kits reported total ammonia and unionized ammonia was calculated from equilibrium constants as described in Emerson et al. (1975). Although ammonia resides in equilibrium with ammonium, calculations verified the order of magnitude difference, and the ecologically relevant concentration of ammonia used in treatments. Exposure lasted 8 days. Concentrations were monitored every 12 h and reset on days three, five, and seven for consistent exposure.

After the isolation week, *O. rusticus* pairs were placed in ten—gallon tanks split into two equal sections with a removable opaque divider for the exposure and behavioral assay trials. An opaque divider prevented visual and chemical signaling between the two crayfish. *O. rusticus* were introduced to each tank for an acclimation period of ten minutes. After 10 min, the opaque divider was removed and a behavioral assay was conducted. The agonistic behavioral assay began when the opaque divider was removed and crayfish were within one body lengths apart. The assay was considered complete after a winner was assigned by one crayfish retreating or tail flipping from the interaction and crayfish were one body length apart for at least 10 s (Fero 2007). All behavioral interactions were video—recorded from above with a Panasonic HDC—HS700K 3MOS Hybrid Full HD 1920 × 1080 60p Camcorder. The first agonistic encounter was used to assign behaviors through a pre—established ethogram (Moore 2007; Table 1).

**Table 1:** Agonistic ethogram used to determine levels of fight intensity as adapted from Moore PA (2007)

Assigned number	Behavior
− 2	Tail flip away from the opponent
− 1	Back away slowly from the opponent
0	Ignore opponent with no response or threat display
1	Slowly approaching opponent, no threat display
2	Approach opponent with a meral spread
3	Boxes and pushes opponent open—clawed
4	Grasps opponent with claws and dances
5	Unrestrained fighting and tearing of appendages

Total fight duration, time to reach intensity levels, and time at various intensity levels from Table 1 were measured. Total fight duration was defined as the time from fight initiation (approaching opponent) to when a winner was assigned. Not all agonistic battles included escalated behaviors (greater than two on the ethogram), therefore we decided to analyze early stages of agonistic interactions (intensity levels 1 and 2. Time to reach intensity levels were defined as the total time from beginning of the behavioral assay to the first-time crayfish exhibited the respective intensity level behavior (Table 1). Time spent at intensity levels were defined as the total amount of time crayfish were at a respective intensity level behavior (Table 1). After the first behavioral assay, the opaque divider was replaced and crayfish were again isolated in the arena. Data were collected for separate agonistic interactions on the eighth day of exposure. Behavioral fights on the eighth day did not include an acclimation period as individuals were already in the behavioral assay arenas. Linear mixed models (LMM) followed by analysis of deviance tables using Type II Wald Chi Square tests (Zuur et al. 2009) were used to determine the effect of exposure and time on fight durations, time to reach different intensities, and time spent at different intensities in the lme4 package (Bates et al. 2015) in R statistical software (version 3.3.0) (R Development Core Team 2016). Models were constructed using time (Day 1 and 8) and ammonia concentration (control, 0.9, and 9) as fixed effects and animal ID as a random effect to account for the repeated measures design. Differences of least squares means (‘difflsmeans’) from the lmerTest package (Kuznetsova et al. 2016) in R was used as a post hoc test for significant differences of the main effects.

## Results and Discussion

**Fig. 1:**
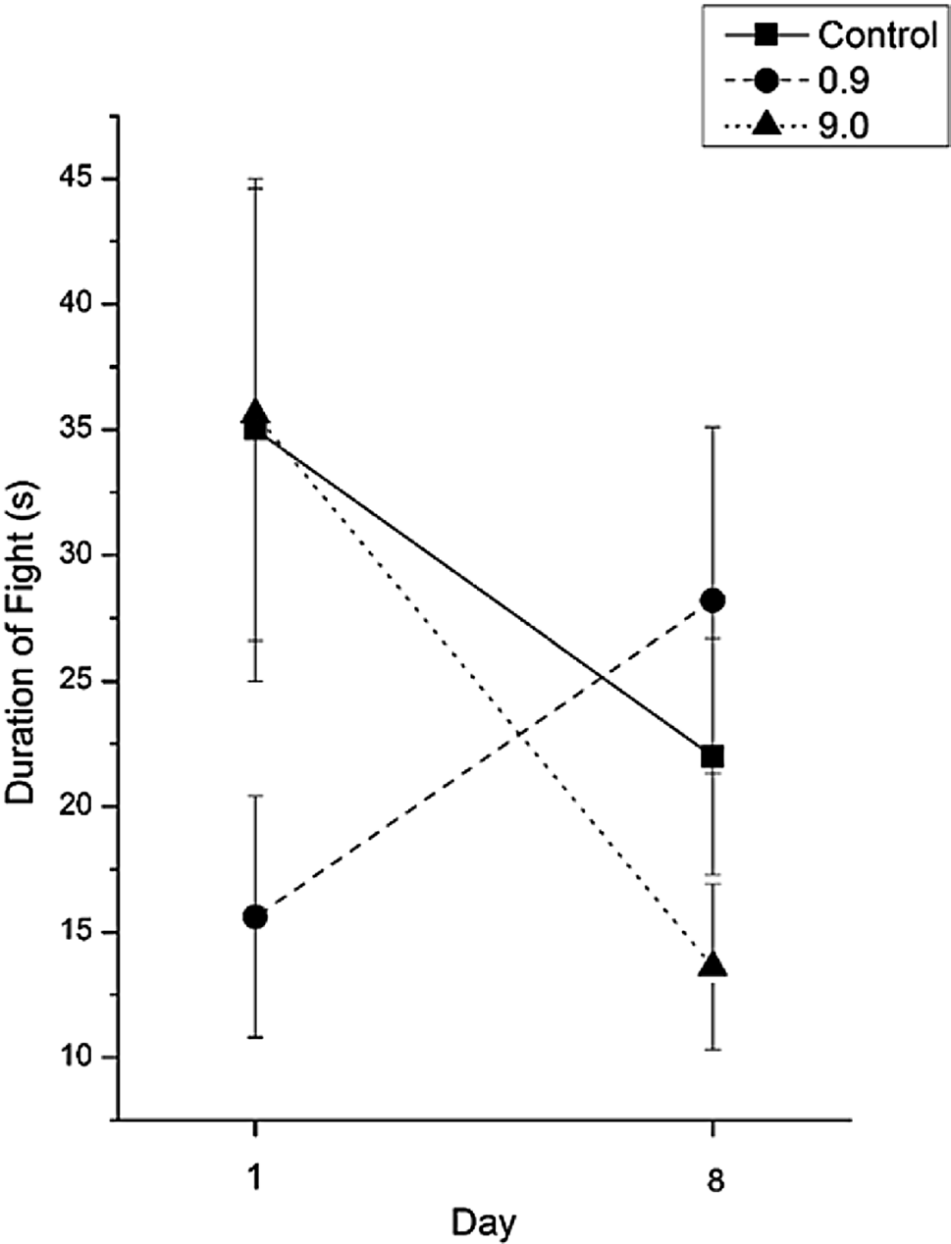
Duration (s ± SE) of agonistic interactions on day one and day eight of ammonia exposure

We observed a significant interaction effect of treatment and exposure length on total fight duration (Fig. 1; X^2^ = 7.72, df = 2, p = 0.021). On day 1 crayfish in the low exposure fought for a significantly shorter duration compared to the control and high treatment. On day eight, crayfish in the low exposure fought for a significantly longer duration compared to the high exposure treatment but no difference compared to control crayfish (Fig. 1). Additionally, crayfish in the high exposure treatment fought for a significantly longer duration on day one than on day eight. Lastly, no statistically significant difference was observed in fight duration of control crayfish between day one and day eight (p > 0.05).

**Fig. 2:**
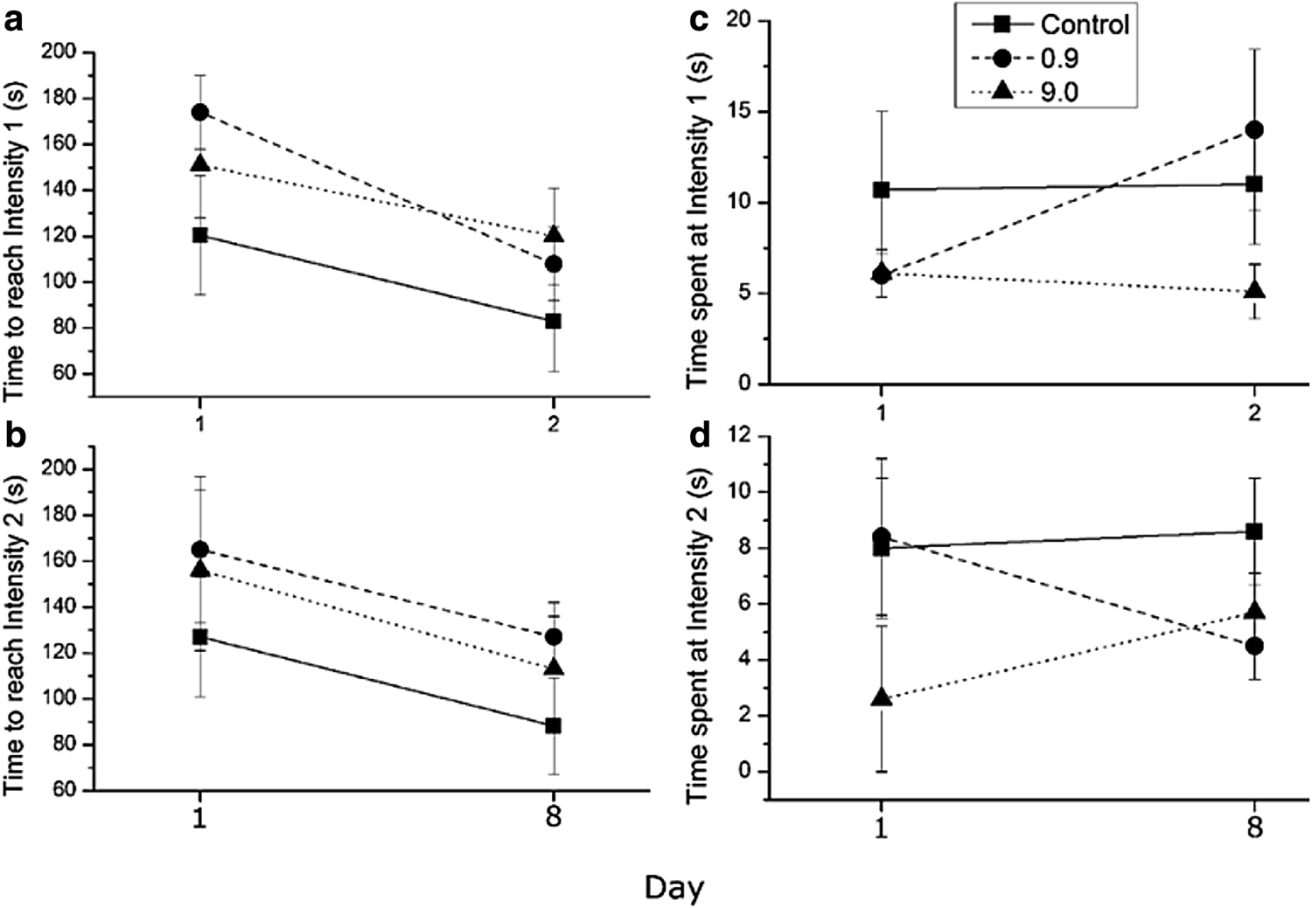
Duration (seconds ± SE) of time to reach (**a**, top left) intensity level 1, and (**b**, bottom left) intensity level 2. Duration (seconds ± SE) of time spent at (**c**, top right) intensity level 1 and (d, bottom right) intensity level 2

Exposure length exhibited a marginal effect on the aggressive escalation of agonistic interactions, measured as the length of time to reach intensity level one (Fig. 2a; X^2^ = 3.65, df = 2, p = 0.16). Crayfish pairs in the low treatment took longer to reach intensity level one on day one than the control pairs on day eight. No difference in the length of time to reach intensity level two was observed (Fig. 2b, p > 0.05).

We observed a significant effect of exposure length on the intensity of agonistic interactions, measured as the amount of time spent at intensity level 2 (meral spread threat display). Crayfish pairs in the high treatment spent significantly more time at intensity level 2 on day one than on day eight. (Fig. 2d; X^2^ = 4.38, df = 1, p = 0.036). No interaction effect of treatment and exposure length was observed for time spent at intensity level 2. A marginal effect of treatment was observed for time spent at intensity level 1 (slowly approaching opponent) (Fig. 2c; X^2^ = 3.65, df = 2, p = 0.161). *O. rusticus* in the low exposure exhibited more time at intensity level one on day eight than on day one. No statistically significant effect of exposure length (X^2^ = 1.13, df = 1, p = 0.288), or the interaction (X^2^ = 2.98, df = 2, p = 0.225) was observed for time at intensity level 1 (Fig. 2).

These results suggest that crayfish fighting behavior is altered in the background presence of sublethal ammonia concentrations. After eight days of exposure to 0.9 and 9.0 mg/L levels of ammonia, duration of fights between crayfish pairs increased and decreased, respectively (Fig. 1). Crayfish pairs exposed to 0.9 mg/L concentration of ammonia exhibited longer time to escalate the agonistic interaction. Crayfish pairs exposed to 9.0 mg/L of ammonia exhibited meral spread threat displays to their opponent for a longer duration on day one than on day eight (Fig. 2). Background presence of chemicals in the environment could affect a crayfish’s ability to extract ecologically relevant information from chemical stimuli in conspecific urine through masking, chemosensory impairment, and/or interruption of physiological processing in the organism (Sutterlin 1974). All three mechanisms are playing a role and we cannot definitively argue for the independent degree of impact each mechanism has in our study. Mechanisms would require further study to parse out the role each plays during agonistic interactions. Our results from exposure to 0.9 mg/L of ammonia could be explained by acute chemosensory impairment.

Chemosensory impairment is a result of temporary damage to the external chemoreceptors as the animal is exposed to external chemicals. The transmittance of chemical information via urine signals is used during assessment stages of fights and with this information absent, fight duration should increase (Zulandt-Schneider et al. 2001; Wofford et al. 2015). If our results indicate chemosensory impairment such that information contained in chemical signals is missing, we would expect agonistic fight duration to increase over the 8 days. The fight duration of the 0.9 mg/L ammonia treatment for day one was significantly shorter than the fight duration of the control group. However, at day eight, there was no significant difference between the low ammonia treatment and the control group (Fig. 1). Average fight duration increased from day one to day eight while no difference was observed for crayfish in the control group. *O. rusticus* in the 0.9 mg/L exposure treatment also spent more time at a lower intensity level on day eight than on day one, indicating prolonged time at a reduced intensity of slowly approaching the opponent. These results together support a hypothesis of reduced chemical transmittance of social information during early stages of an agonistic battle. Results from the 9.0 mg/L ammonia treatment could be explained by physiological disruption of internal information processing.

If the 9.0 mg/L ammonia treatment led to slight internal physiological changes (Harris et al. 2001), we would expect crayfish to reduce agonistic behavior. Internal processing of information is lost or hindered from the breakdown of neurological function. Fighting pairs of the 9.0 mg/L ammonia treatment showed reduced aggression and total fighting duration on day eight compared to day one (Figs. 1, 2). *O. rusticus* in the 9.0 mg/L treatment exhibited longer time at using threat displays on day one than day eight prior to engaging in a fight, indicating a potential reluctance or reduced ability to fully engage in an agonistic battle. While masking also reduces the ability to acquire social information, our results do not solely support the mechanism of masking.

We cannot rule out masking as an impairment mechanism, however our results do not suggest masking as the main mechanism underlying observed behavioral differences. Each crayfish had an acclimation time before the first behavioral assay. The agnostic/antagonistic binding of molecules to sensory receptor sites during acclimation would result in extended fight duration because of reduced chemosensory information during agonistic fights. In addition, prolonged exposure to social odors has been shown to increase fighting times (Bergman et al. 2005). Therefore, if masking were the sole mechanism, fight durations would be significantly longer in both treatments than the control on day one and day eight. This was not observed (Fig. 1). Alteration in the daily behavior of *O. rusticus* from reduced ability to obtain ecological information from either mechanism can have cascading environmental impacts. It is possible that these effects could be short term if the crayfish’s physiology acclimates or desensitizes to the presence of increases ammonia concentrations.

Two exceptionally important behaviors mediated by chemical stimuli for *O. rusticus* are foraging and mating. Crayfish utilize chemical cues to orient and locate food but foraging ability is reduced when chemosensory ability is inhibited by atrazine and copper (Belanger et al. 2016; Lahman and Moore 2015). Urine signals from female crayfish are used to communicate mating receptivity and when female urine signals are blocked male courtship behavior is prevented (Berry and Breithaupt 2010). While in reproductive status, male *O. rusticus* use major chelae to discriminate female odors and reduced ability to detect these odors could alter crayfish population dynamics (Belanger and Moore 2006). *O. rusticus* rely heavily on chemical stimuli composed of ammonia for social behaviors. Our study adds to knowledge of anthropogenic related impacts to organisms.

## References

[CR1] Aquiloni L, Gonçalves V, Inghilesi AF, Gherardi F (2012). Who’s what? Prompt recognition of social status in crayfish. Behav Ecol Sociobiol.

[CR2] Arthur JW, West CW, Allen KN, Hedtke SF (1987). Seasonal toxicity of ammonia to five fish and nine invertebrate species. Bull Environ Contam Toxicol.

[CR3] Bates D, Maechler M, Bolker B, Walker S (2015). Fitting Linear Mixed-Effects Models Using lme4. J Stat Softw.

[CR4] Belanger RM, Moore PA (2006). The use of the major chelae by reproductive male crayfish (*Orconectes rusticus*) for discrimination of female odours. Behaviour.

[CR5] Belanger RM, Mooney LM, Nguyen HM, Abraham NK, Peters TJ, Kana MA, May LA (2016). Acute atrazine exposure has lasting effects on chemosensory responses to food odors in crayfish (*Orconectes virilis*). Arch Environ Contam Toxicol.

[CR6] Bergman DA, Moore PA (2005). The role of chemical signals in the social behavior of crayfish. Chem Senses.

[CR7] Bergman DA, Moore PA (2005). Prolonged exposure to social odours alters subsequent interactions in crayfish (*Orconectes rusticus*). Anim Behav.

[CR8] Bergman DA, Martin AL, Moore PA (2005). Control of information flow through the influence of mechanical and chemical signals during agonistic encounters by the crayfish, *Orconectes rusticus*. Anim Behav.

[CR9] Berry FC, Breithaupt T (2010). To signal or not to signal? Chemical communication by urine: borne signals mirrors sexual conflict in crayfish. BMC Biol.

[CR10] Breithaupt T, Eger P (2002). Urine makes the difference: chemical communication in fighting crayfish made visible. J Exp Biol.

[CR11] Dusenbery DB (1992). Sensory ecology: how organisms acquire and respond to information.

[CR12] Eddy FB (2005). Ammonia in estuaries and effects on fish. J Fish Biol.

[CR13] Emerson K, Russo RC, Lund RE, Thurston RV (1975). Aqueous ammonia equilibrium calculations: effects of pH and temperature. J Fish Res Board Can.

[CR14] Endler JA (1987). Predation, light intensity and courtship behaviour in *Poecilia reticulata* (Pisces: PoecUiidae). Anim Behav.

[CR15] Endler JA, Amundsen T, Rosenqvist G (2000). Evolutionary implications of the interaction between animal signals and the environment. Animal signals: signaling and signal design in animal communication.

[CR16] Fero K, Simon VJ, Moore PA (2007). Consequences of social dominance on crayfish resource use. Behaviour.

[CR17] Gelowitz CM, Mathis A, Smith RJF (1993). Chemosensory Recognition of Northern Pike (*Esox lucius*) by Brook Stickleback (*Culaea inconstans*): population differences and the influence of predator diet. Behaviour.

[CR18] Harris RR, Coley S, Collin S, McCabe R (2001). Ammonia uptake and its effects on ionoregulation in the crayfish *Pacifastacus leniusculus*. J Comp Physiol B.

[CR19] Hazlett BA (1985). Chemical detection of sex and condition in the crayfish *Orconectes virilis*. J Chem Ecol.

[CR20] Jurcak AM, Moore PA (2014). Behavioral decisions in sensory landscapes: crayfish use chemical signals to make habitat use choices. J Crustac Biol.

[CR21] Kuznetsova A, Brockhof PB, Christensen RHB (2016) lmerTest: tests in linear mixed effects models: R Package version 2.0–33, R Foundation for Statistical Computing, Vienna. https://cran.r-project.org/web/packages/lmerTest/index.html

[CR22] Lahman SE, Trent KR, Moore PA (2015). Sublethal copper toxicity impairs chemical orientation in the crayfish, *Orconectes rusticus*. Ecotox Environ Safe.

[CR23] Moore PA (2007). Agonistic behavior in freshwater crayfish: The influence of intrinsic and extrinsic factors on aggressive encounters and dominance.

[CR24] Moore PA, Crimaldi J (2004). Odor landscapes and animal behavior: tracking odor plumes in different physical worlds. J Mar Syst.

[CR25] Ohio Environmental Protection Agency (2010) Appendices to biological and water quality study of the Portage River basin, select Lake Erie tributaries, and select Maumee River tributaries, 2006–2008. Division of Surface Water

[CR26] Pavey CR, Fielder DR (1996). The influence of size differential on agonistic behavior in the freshwater crayfish, *Cherax cuspidatus* (Decapoda: Parastacidae). J Zool.

[CR27] R Core Development Team (2016). R: a language and environment for statistical computing. – R Foundation for Statistical Computing, Vienna. https://www.R-project.org/

[CR28] Romano N, Chaoshu Z (2010). Survival, osmoregulation, and ammonia - N excretion of blue swimming crab, *Portunus pelagicus*, juveniles exposed to different ammonia - N and salinity combinations. Comp Biochem Physiol C.

[CR29] Sutterlin AM (1974). Pollutants and the chemical senses of aquatic animals: perspective and review. Chem Sen.

[CR30] Weissburg MJ, Zimmer-Faust RK (1994). Odor plumes and how blue crabs use them in finding prey. J Exp Biol.

[CR31] Wofford SJ, Earley RL, Moore PA (2015). To fight or not to fight? Male and female crayfish decide differently when engaged in mixed sex interactions. Behavior.

[CR32] Wolf MC, Martin AL, Simon JL, Bergner JL, Moore PA (2009). Chemosensory signals in stream habitats: implications for ecological interactions. J North Am Benthol Soc.

[CR33] Young-Lai WW, Charmantier-Daures M, Charmantier G (1991). Effect of ammonia on survival and osmoregulation in different life stages of the lobster *Homarus americanus*. Mar Biol.

[CR34] Zulandt-Schneider RA, Huber R, Moore PA (2001). Individual and status recognition in the crayfish, *Orconectes rusticus*: the effects of urine release on fight dynamics. Behaviour.

[CR35] Zuur AF, Ieno EN, Walker NJ, Saveliev AA, Smith GM (2009). Mixed effects models and extensions in ecology with R.

